# Habitat Suitability and Establishment Limitations of a Problematic Liana

**DOI:** 10.3390/plants10020263

**Published:** 2021-01-29

**Authors:** Christopher C. Dickinson, John G. Jelesko, Jacob N. Barney

**Affiliations:** School of Plant and Environmental Sciences, Virginia Tech, Blacksburg, VA 24061, USA; chrisd1@vt.edu (C.C.D.); jelesko@vt.edu (J.G.J.)

**Keywords:** deer, herbivory, poison ivy, *Toxicodendron radicans*

## Abstract

The US native liana, poison ivy *(Toxicodendron radicans*), responsible for contact dermatitis in humans, is a competitive weed with great potential for expansion in disturbed habitats. To facilitate a better understanding of this threat, we sought to evaluate habitat suitability, population demography, and biotic interactions of poison ivy, using a series of complementary field studies in the two habitats where it most commonly occurs—forest interiors and edges. Of the 2500 seeds planted across both habitats, poison ivy initially colonized forest interiors (32% emergence) at a higher rate than edge habitats (16.5% emergence). However, forest interior seedlings were less likely to survive (interior *n* = 3; edge *n* = 15), which might be attributed to herbivore pressure when the seedlings were smaller in the less competitive forest interior. Once established, the poison ivy seedlings appeared to be more tolerant of herbivory, except that of large grazers such as deer. The early life stage of seedling emergence, survival, and establishment are critical in poison ivy success, with biotic pressure, especially from plant competition and deer, limiting recruitment. A suitable habitat of this expanding native liana would increase with increasing forest fragmentation, but might be buffered by the expanding deer population.

## 1. Introduction

Lianas, or woody vines, play an important role in forests as competitors of their tree hosts [1,2]. As structural parasites, lianas are able to allocate more resources towards the production of leaves, leading to a myriad of advantages, including increased competition for light and structural damage to their host’s integrity [3]. Some lianas can also creep along the ground with offshoots or stolons from a main vine that smother slower growing understory seedlings [4]. These advantages are further facilitated by increased atmospheric CO_2_ levels, as lianas accumulate relatively more biomass than other woody plants [5,6,7]. Additionally, increased forest fragmentation and management practices increase the overall disturbance, and also increase the abundance of forest edges, both of which benefit liana recruitment and establishment [8].

At an ecosystem level, lianas can have dramatic impacts. For example, kudzu (*Pueraria lobata*) is capable of producing vast dense mats covering many hectares comprising tens of thousands of individual kudzu plants [9]. Uncontrolled for over a century, in Mississippi alone, kudzu accounts for an estimated $54 million USD per year in timber losses [10]. In tropical forests, native lianas are associated with reductions in net forest primary-production [11,12]. Van de Heijden et al. [2] found that tropical forests might be capturing up to 76% less carbon per year than possible due to increased tree mortality attributed to lianas. Increasingly, we are seeing evidence that some native lianas are responding favorably to human-mediated land-use and environmental change. Some native species exhibit similar ecological and economic impacts, as well as the spread and abundance associated with the non-native invaders [13,14]. For example, the native *Vitis* spp. in the US alter the community structure and fire regimes in temperate forests. However, gaps remain on how native temperate lianas respond to drivers or limitations of range expansion, including biotic and abiotic filters.

Range expansion is classically considered to occur in four stages—transportation, colonization, establishment, and spread [15]. Colonization, the initiation of new plants from propagules in a new location and establishment, or the long-term survival of new populations, is also applicable to range-expanding natives. Unlike introduced non-native plants that are transported across large distances, native plants are already present, or are nearby, and are much more likely to experience the local conditions. Native species might either expand their range into nearby novel landscapes, or become locally abundant, as a result of disturbances or other anthropogenic changes [14]. However, questions remain about what aspects of seed dispersal, emergence, establishment, and survival facilitate new population establishment? It is well known that biotic (e.g., seed predators and herbivores) and abiotic (e.g., light availability) factors influence the success of species colonizing new locations [15]. However, we currently lack basic ecological and population demographic information on most temperate lianas. This information will become increasingly important to understand shifting species geographic distributions, and how plant communities respond to global change, especially anthropogenic disturbance and land-use change.

One such liana that positively responds to landscape and global change is *Toxicodendron radicans* (L) Kuntze (poison ivy), a dioecious perennial native to the US, which is best known for its capacity to cause contact dermatitis in humans, due to the chemical urushiol found throughout the plant [16,17]. Due to poison ivy’s emerging role as a weedy species, it is a regulated noxious weed in Minnesota, Ontario, Quebec, and Manitoba [18,19]. Additionally, poison ivy appears to favor anthropogenically disturbed and edge habitats [20,21,22], which are also likely to continue to become more common with urbanization and habitat fragmentation [23]. However, we lack basic information of the conditions that facilitate poison ivy seed germination, population establishment, and biotic interactions (i.e., seed and seedling herbivory), which are needed to improve our understanding of how this species might expand its range in the future.

Here, we aim to facilitate a better understanding of the biotic and abiotic limitations of poison ivy at the early stage of range expansion; namely seed and seedling. Specifically, we address the following key questions using several complementary field experiments—(1) Do drupes that pass through a simulated animal gut establish at higher rates than the untreated drupes?; (2) Does seedling establishment vary between forest edge and interior habitats?; and (3) What impacts do seed predation and seedling herbivory have on poison ivy establishment? This work aims to not only provide key insights into the life history of a problematic and expanding native liana, but also to serve as a foundation to understanding the establishment of temperate lianas.

## 2. Results

### 2.1. Drupe Predation

There were no significant differences in drupe predation whether large herbivores were excluded or not (X^2^ = 0.106, *p* = 0.745). Overall, few drupes were removed by seed predators. In one instance, 19 of 25 drupes were removed, the next highest amount was the removal of 7 drupes, with an average of 3.2 (± 2.3). The high herbivory event was to an unprotected dish and was a rare instance of seed predation in our study.

### 2.2. Seedling Establishment of Poison Ivy in Forest Interior and Edge Habitats

**Figure 1:**
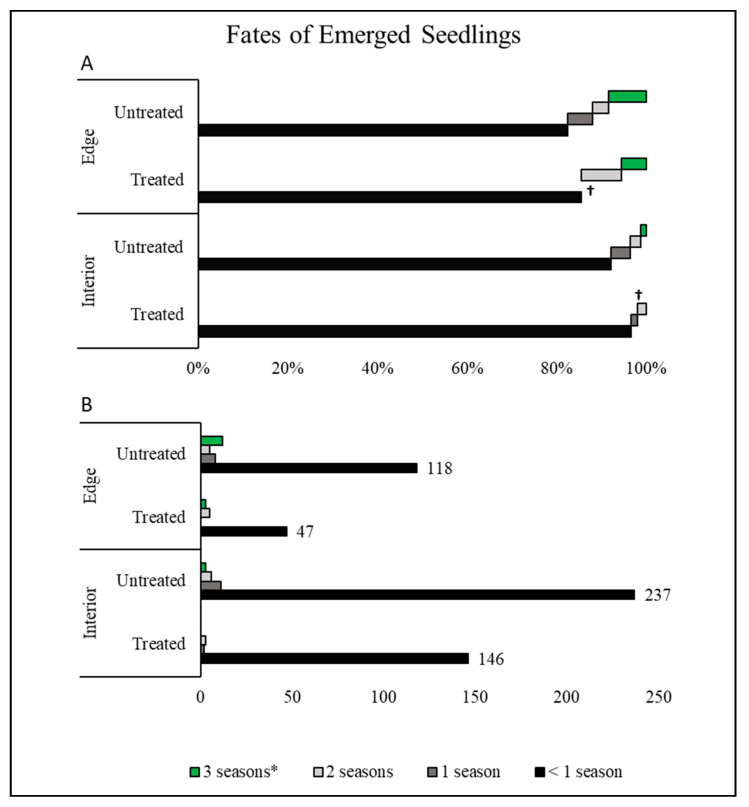
Distribution of poison ivy (*Toxicodendron radicans*) seedling fates for seeds that were “treated” or “untreated”, grown in either the forest edge or interior, represented as (**A**) percentage of total within treatment and (**B**) raw counts (floating values by the bars). Drupes that did not produce seedlings were excluded. A cross (†) indicates that there were no plants whose fate placed them within that category. An asterisk (*) signifies poison ivy plants that survived the duration of the experiment.

Seedling emergence from drupes treated to simulate bird gut passage began in early to mid-June, 26 days after planting, while the untreated drupes did not emerge until the beginning of July, 41 days after planting. Despite the later start, the untreated drupes resulted in two-fold as many seedlings as the treated drupes (Figure 1, Table 1). Nearly twice as many poison ivy seedlings emerged in the forest interior than the edge habitat by the end of the first season (black bars in Figure 1B). Additionally, more plants emerged in plots with more bare ground and acidic soils (Table 1). Overall, seedling emergence past the first year was exceedingly rare (*n* = 11, 1.8%, gray bars in Figure 1).

While 608 seedlings resulted from 2500 planted drupes (24%), only 3% (18 total plants, green bars in Figure 1) survived until the end of the study (3 seasons). Most seedlings did not survive beyond two months after emergence, with 53 plants (8.8%) remaining at the beginning of the first winter, with another 17 plants not surviving the first winter. In most cases, plants that died in the first year did not show evidence of abiotic stress such as wilting or nutrient deficiency. Rather, seedlings, particularly at the cotyledon stage, were simply missing entirely, likely as a result of herbivory, as was apparent on some seedlings that did survive. Plants in edge habitats were much more likely to survive in each year of the study (Figure 1, Table 1). This resulted in more surviving plants in the edge habitat despite the 2:1 advantage in initial emergence rates within the interior habitat. Once established, the chances of survival did improve in the second and third years, with a 64% survival rate in the second year and a 49% survival rate in the third. The remaining plants were 12.5 cm (± 3.6) tall, with 2.6 (± 1.9) leaves, and 0.33 g (± 0.17) aboveground biomass; however, not enough plants survived to statistically evaluate the differences between drupe treatments or habitats.

### 2.3. Seedling Herbivory Tolerance

**Figure 2:**
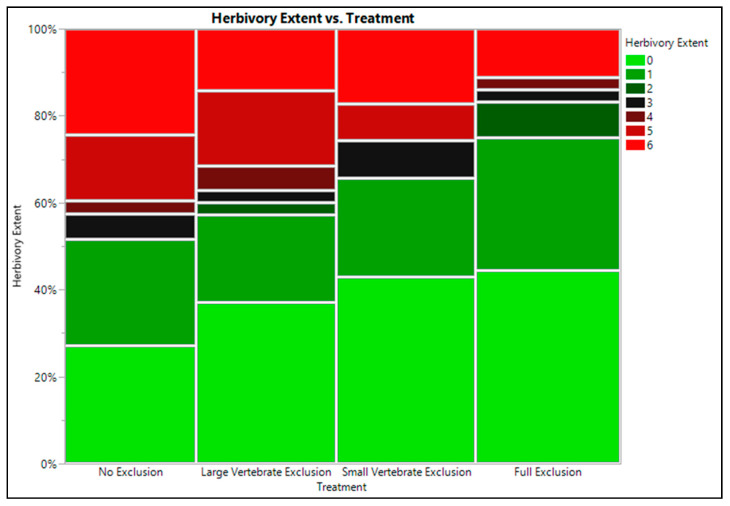
Distribution of herbivory extent classification for each event occurring on poison ivy (*Toxicodendron radicans*) plants in each of our four herbivore-exclusion treatments—no exclusion; large vertebrate exclusion only; small mammal exclusion that also included large mammal exclusion; and full exclusion (no herbivory). Herbivory severity was classified according to the scale—(0) no herbivory, (1) low, classified as 1–25% of a leaf removed; (2) moderate, as 25–50% of a leaf removed; (3) high, as 50–99% of a leaf removed, (4) very high, as 1 full leaf removed, (5) severe, as 2 or more full leaves removed, or (6) death of the plant.

**Figure 3:**
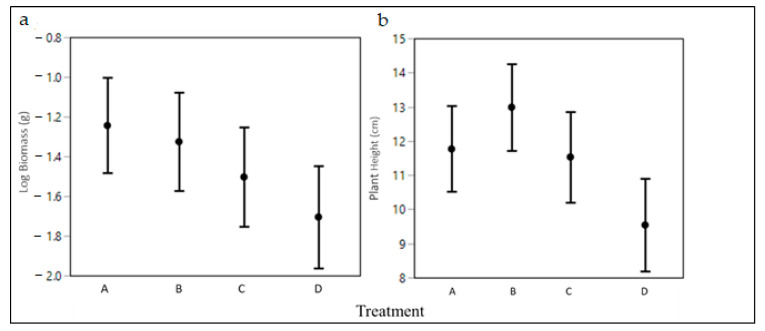
Growth metrics of poison ivy (*Toxicodendron radicans*) plants after two years growing in varying levels of herbivore protection: (A) full exclusion (no herbivory), (B) small mammal exclusion that also included large mammal exclusion; (C) large mammal exclusion only; and (D) no exclusion. (**a**) Natural log biomass across exclusion treatments (LS means ± 95% CI’s). (**b**) Plant height across exclusion treatments (LS means ± 95% CI’s).

Seedling survival across all treatments (high, medium, low, and no exclusion) was high (82.5%), and did not vary among treatments (Table 2). The incidence of disease and herbivory did not vary among the exclusion treatments (Table 2). Extent of herbivory was >2-fold higher with no herbivory exclusion as compared to the full exclusion treatments (Figure 2, Table 3), though exclusion treatment was not a strong predictor for herbivory extent overall (X^2^ = 5.3, *p* = 0.147). Poison ivy final biomass varied among exclusion treatments (*p* = 0.055), with plants open to all herbivores accumulating an average of 32% less biomass than the herbivore excluded plants. Plants in the open plots were also shorter by 2.6 cm (27%, Figure 3, Table 4).

## 3. Discussion

As structural parasites, lianas play an important role in forests as competitors [1,2], and increasingly we are understanding that range-expanding native temperate vines can also cause negative impacts [13], especially in response to anthropogenic drivers of environmental and land-use change [24]. We evaluated the early life stage of establishment success of the problematic native liana poison ivy to better understand habitat suitability and the role of biotic limitations to that success.

The transport and spread of poison ivy propagules can occur through avian endozoochory of the drupes [25,26] and gravity dispersal below female vines. Due to its thick fleshy mesocarp and water-impermeable endocarp, mechanical and chemical drupe scarification leads to higher germination rates in the lab [27], though Penner et al. [28] found that avian digestion does not increase poison ivy seedling germination. Our study demonstrates that simulated avian endozoochory is not a prerequisite for germination and subsequent seedling emergence in the field. We found that untreated (raw) drupes produced seedlings at higher rates than those that were mechanically and chemically scarified, which contradicted previous laboratory studies that found enhanced germination of the “treated” versus “raw” drupes [27,28]. Benhase and Jelesko [27] observed that drupe treatment with sulfuric acid led to pitting of the endocarp, which they suspected allowed the embryo to imbibe water and subsequently germinate. This pitting might have resulted in germination under less favorable conditions (i.e., inadequate moisture leading to embryo desiccation in the drupe) in our field experiment, leading to failed seedling emergence. Alternatively, the treated drupes might have been compromised due to the decreased defenses in the soil [29,30]. However, we also found a higher seedling emergence rate in more acidic soils, possibly due to more complete or faster degradation of the mesocarp, endocarp, brachysclereid, or osteosclereid layers of the endocarp of the untreated drupes. Our work suggests that avian digestion is not required for successful recruitment.

Once drupes are dispersed, successful establishment is contingent on both abiotic and biotic interactions [15]. Aside from microbial degradation, seed predation is the most common biotic filter limiting germination [31]. For example, Penner et al. [28] noted that squirrels act as seed predators of Rydberg’s poison ivy, crushing the drupes in their teeth and extracting the embryos, rendering them non-viable. Our evaluation of post-dispersal seed predation suggests squirrels and other small rodents that could climb over our exclusion fences, or birds were predating poison ivy drupes. However, we observed relatively low seed predation (~12%), suggesting that under our conditions poison ivy drupes experienced little biotic resistance, following dispersal and preceding germination. Further, secondary dispersal might result from attempted seed predation documented in other systems [32], offsetting the perceived loss in propagule pressure. Further, in the seed introduction experiment we observed very few new seedlings after the first year, suggesting either the seeds germinated and died immediately, were dormant, or were eaten by seed predators. The former we could not observe, and the latter was not supported by our seed predation experiment, suggesting that poison ivy might possess long-term physical dormancy. A better understanding of both processes is necessary, however, to fully understand poison ivy population demography.

Once propagules are dispersed to a new location and the conditions for germination are met, the seedlings now face biotic and abiotic limitations to establishment. Poison ivy is capable of readily colonizing areas after fire disturbance, can persist in a wide range of soil types [33], and has a broad host tree suitability [34]. An observational study conducted by Gillis did not find higher incidence of poison ivy in forest edge habitats [33]. However, other surveys contradict this [21,22], especially in instances when disturbance create edge habitat [20,35,36]. Ultimately, all evidence to date of higher poison ivy abundance in edge habitats were observational; thus, our work represents the first manipulative investigation of forest edge vs. interior habitat suitability for poison ivy colonization.

We found an interesting “switch” in habitat preferences between poison ivy life history stages. Seedling emergence was highest in habitats where resident plant competition was lowest in the forest interior. However, while emergence rate was initially much higher in the forest interior, seedling survival was highest in edge habitats where plant cover was higher. The low survival in the interior habitat did not appear to be due to disease or abiotic stress; rather the low survival was most likely due to herbivory. The forest edge in our system had very high plant cover of grasses, forbs, and shrubs, while the understory had sparse cover of understory plants and occasional shrubs—both typical of deciduous forests in the US. Thus, while less competition was beneficial for emergence, being hidden within other plants might have afforded long-term survival by eluding herbivores. Previous surveys [20,33,35,36] did not explicitly evaluate differences in life stages, while we found that life stage is an important consideration. While our study did not explicitly evaluate disturbance, edge habitats often occur as the result of disturbance such as canopy gaps, following tree death [20,35,36]. This pattern was observed in many range expanding native plants, especially after disturbances such as changes in fire regimes [14]. Poison ivy demonstrated higher abundances following both canopy collapse [20], and post-fire recolonization [33].

Surprisingly few poison ivy plants survived until the end of the study. Of the 2500 drupes planted and subsequent 608 seedlings that were found, only 18 individuals survived all three years. However, this extremely low survival rate (3%) could be offset in several ways. First, poison ivy is capable of reproducing clonally through either rhizomes or stolons. The capacity for poison ivy to reproduce in this manner was not evaluated, although our personal observations of the sheer number of plantlets found under or near an older liana suggests that this could be a major life history strategy. Also a mature female poison ivy liana is capable of producing many drupes over its lifespan [33]. Thus, a second method to overcome the observed low establishment rate is through the production of many drupes. In other words, like many plants, poison ivy hedges against seedling mortality through other life history strategies.

Establishment is often considered to be primarily limited by biotic interactions [15]. Herbivory of poison ivy can be significant, especially in winter months, when other food sources are scarce [25,28,33]. Urushiol has no apparent effect on non-human mammalian species; with goats, deer, and cattle observed feeding on poison ivy [25]. Several arthropods are known to feed on the leaves of poison ivy [37]. The breadth of vertebrate and insect herbivory suggests that urushiol is not involved in herbivory defense, at least not as a general defense. Despite observational reports of various herbivores on poison ivy, there exists no empirical evidence of the identity and impact of herbivores on poison ivy establishment [25]. Averill et al. [38] found in a meta-analysis that poison ivy presence is a moderate indicator of deer exclusion, suggesting that deer do browse poison ivy. In contrast to our seed emergence and establishment study, survival in our herbivore exclusion study was quite high at ~80%, even in the unprotected plots. This, compounded with higher survival rates in the later years of the establishment study, suggests that young seedlings are most susceptible to herbivory in the first few weeks/months, following germination. The transplants used in the herbivore exclusion study were approximately two months post germination and were grown under ideal laboratory and greenhouse conditions. Perhaps these favorable conditions enabled the plants to lignify more quickly, providing additional chemical defenses, or were simply large enough to tolerate browsing. However, the exact mechanism(s) for differential survival at this critical life stage requires further investigation.

While survival was high in the exclusion study, plants open to all herbivores experienced more severe tissue loss and accumulated less biomass than plants with even minimal protection. As is common in eastern deciduous forests, deer were quite numerous, as observed on the game cameras, and we suspect them to be the primary herbivores responsible for the herbivory of unprotected plants. Our trail cameras observed many animals in the area, but we only captured one clear incidence of deer browsing poison ivy, but were unable to gather additional video evidence to estimate their overall effect. Deer are also capable of completely removing seedlings, as we assumed in the establishment study—though we lack video evidence to confirm this. Insect herbivory was common, as we noted many instances of small removal of leaf material. Insect herbivores of poison ivy are numerous and span many families [25,39], and our chosen insecticide might not have adequately covered the breadth of these insects. This might explain why our insecticide treatment did not fully control insect herbivory.

Our results suggest that the early life stage of seedling emergence and establishment are critical life history transitions in poison ivy population demography. We observed minimal seed predation, and once seedlings were established, they appeared to be tolerant of most herbivory. We expected an increase in poison ivy abundance, as more individuals were able to escape herbivore pressure by climbing into the canopy. As observed in tropical forests, lianas were quite capable of competing with their tree hosts [12]. Native lianas like poison ivy represent a relatively under-studied threat to temperate forests and human health. We demonstrated how poison ivy, and likely other lianas, were able to colonize disturbed habitats, while also being hindered by large mammals such as deer at early life stages. Liana abundance dramatically increased in recent decades, presumably through habitat fragmentation and land-use changes [1]. However, range expansion of poison ivy might be buffered by increasing deer populations in the Eastern US, resulting in a complex set of interacting factors shaping poison ivy demography.

## 4. Materials and Methods

To address our objectives, we conducted several complementary experiments, which we detail below in order of life stage—seed, seed emergence, and seedling survival and establishment. All experiments were conducted at the Kentland Research Farm near the Virginia Tech campus (37.197111, −80.581916) in Whitethorne, VA, USA.

### 4.1. Post-Dispersal Drupe Predation

Poison ivy drupes are produced on female vines, from summer to fall. Drupes for this experiment were collected from the Virginia Tech Golf Course (37.227841, −80.432291) in the fall of 2015. This study was designed to identify the fate of the exposed poison ivy drupes to predation. On 7 August 2018, drupes were placed in 9.5 cm^2^ plates constructed of 0.635 cm mesh hardware cloth and lined with shade cloth. Plates were buried in the ground up to the lip of the plates (~1 cm), with 25 drupes per plate. One plate of drupes was placed either within 0.625 cm mesh hardware cloth (we used the small animal exclusion plots from below), to exclude small and large vertebrates, or in open plots. This was repeated in three blocks at three sites (3 sites × 3 blocks × 2 treatments = 18 dishes each with 25 drupes: 25 × 18 = 450 total drupes). In other words, there were 9 dishes of 250 total drupes either in the open or in a fence to prevent vertebrate access. After one week, the plates were collected and the remaining drupes counted.

#### Analysis

A generalized linear model with a Poisson distribution and a log-link function with site and exclusion and their interaction as fixed effects was used to analyze the numerical count data of missing drupes using JMP 13 Pro (SAS Institute Inc., Cary, NC, USA).

### 4.2. Poison Ivy Seedling Establishment in Forest Interior and Edge Habitats

#### 4.2.1. Plant Material

Drupes from the same population above were either left untreated (“Untreated”) or mechanically and chemically scarified (“Treated”) [27]. One modification was made to the Benhase protocol—rather than washing the residual bleach from the drupes with water, the drupes were instead immediately plated onto 0.5× MS media plates, ensuring sterility rates approaching 100%. The acid “treated” drupes were included to simulate a drupe that was bird dispersed, which was thought to be the primary dispersal mechanism of poison ivy, having the exocarp, mesocarp, and the endocarp brachysclereid and osteosclereid cell layers partially removed [27,28]. Thus, we tested whether recruitment varied among drupes that were gravity (“untreated”) or bird (“treated”) dispersed.

#### 4.2.2. Sowing

In addition to testing for drupe dispersal effects, as outlined above, we were also interested in testing whether poison ivy emergence and establishment varied among the edge and interior forest habitats, as these were the two most common habitats in which poison ivy occurred. In our study, the forest edges comprised the transition from forest to open field or pasture, while forest interior plots were always >15 m from the forest edge. Five replicate sites were chosen that had both an interior and an edge habitat. Within each habitat, we used a stratified random design of 10 1 m^2^ plots, with five plots receiving untreated and five plots receiving treated drupes. In each plot, 25 drupes were gently pressed into the soil in a 5 × 5 grid spaced 20 cm apart, with a 20-cm long bamboo skewer marking the placement of each drupe. In total, 2500 drupes (5 sites × 2 habitats × 2 treatments × 5 plots × 25 drupes) were planted on May 18–20 2016. In other words, we added 625 untreated (raw) and 625 treated drupes in both the forest edge and the forest interior habitat. The sites were not modified or disturbed in any way.

#### 4.2.3. Data Collection

Seedling emergence and survival was tracked weekly from May–August and then biweekly from September–October 2016. In the following years (2017–2018), data collection began once the plants broke dormancy (~April) and concluded in October, or until the leaves senesced. In July–August of each year, percent light transmittance using an AccuPAR model LP-80 ceptometer, soil moisture using a Dynamax TH300 probe, and percent bare ground was collected per plot. Soil samples were collected on 26 August 2016 for each habitat and analyzed by the Virginia Tech Soil Testing Lab. At the end of the third growing season (2018), we recorded plant height and leaf number, and cut all aboveground biomass at the soil surface, dried at 70 °C for one week, and weighed for the final biomass of all remaining plants.

#### 4.2.4. Analysis

Total seedling emergence data from 2016 was modeled using a logistic regression with habitat, treatment, site, and the interaction between habitat and treatment as fixed effects. Additionally, to evaluate the effect of plot-level abiotic factors, emergence data were converted to a percentage at the plot scale, followed by a reverse stepwise linear regression model selection. The initial full model included the following predictors—treatment, percent bare ground, percent soil moisture, soil pH, and their full factorial interactions, as well as site. Percent emergence was arcsine square root transformed to meet model assumptions. Percent bare ground and percent light transmittance were positively correlated (Pearson correlation 0.698, *p*-value = <0.0001), so only the metric of bare ground was included. Higher order non-significant effects were removed step-wise until no non-significant interactions remained. Survival data were modeled using a logistic regression containing habitat, treatment, and site as fixed effects, as well as the interaction between habitat and treatment. Few remaining plants at the end of the study (*n* = 18) necessitated that Firth bias-adjusted estimates were used. All analyses were performed using JMP 13 Pro (SAS Institute Inc., Cary, NC, USA).

### 4.3. Seedling Herbivory Tolerance

#### 4.3.1. Field Sites

This study was designed to isolate the effects of various sized herbivores on poison ivy seedlings. The herbivore exclusion plots were constructed in the forest interior at three of the sites described above, at least 25 m from the interior forest establishment plots. Each site contained three replicate blocks of unmanipulated vegetation in which transplanted poison ivy seedlings were protected from herbivores of various sizes. Each block consisted of four 1.5 m^2^ adjacent plots laid out in a 2 × 2 square, within which the poison ivy seedling were transplanted into the central 1 m^2^. The plots were randomly assigned to one of four treatments—(1) no exclusion, or all herbivores allowed; (2) exclusion of large vertebrates; (3) exclusion of large and small vertebrates; and (4) exclusion of all vertebrate herbivores and insects. Treatment 1 was outside all fencing and thus available to all herbivores. Surrounding treatments 2–4 was 2.1-m tall deer fencing with a 1.3-cm mesh size, in order to exclude deer and other large mammals. For treatment 2, we cut two 25.4 cm holes at the ground level, allowing smaller animals such as rabbits and rodents to enter, while excluding large animals. For treatments 3–4, a secondary fence of hardware cloth with a 0.635 cm mesh size was buried 30 cm, to exclude all vertebrates, but to allow insects. Lastly, plants in treatment 4 were sprayed to wet monthly with the insecticide Sevin (carbaryl), a mix of 120 mL product per liter of water (TechPac LLC, Atlanta, GA, USA) to prevent insect herbivory. Insecticide treatments were made in the morning on rainless days, with the surrounding forest serving as adequate wind protection.

#### 4.3.2. Transplanting

Drupes from the same collection as above were propagated in growth chambers, as previously described by Benhase and Jelesko [27]. After approximately seven weeks, the plants were transferred to a greenhouse for one week to reduce transplant shock, and then planted in the field on 10 July 2017, with five seedlings transplanted per plot. Transplants were organized with one at each of the four corners with the fifth in the center. Transplants that died within five days of planting were replaced. In total, we transplanted 5 seedlings in each plot, replicated in three blocks across three sites. This resulted in 45 poison ivy seedlings in each of the four treatments.

#### 4.3.3. Data Collection

Prior to planting, we recorded height and leaf number (3–6) of each poison ivy seedling to account for size asymmetries at planting. Survival and any noticeable leaf damage were recorded weekly for each individual. Leaf damage was initially classified as caused by either disease or herbivores, and then the extent of that damage was recorded either as a percentage of a single compound leaf, or in the case of full removal, a count of missing leaves. This was conducted on the same schedule as the establishment study and culminated in October 2018. At the conclusion of the experiment, 65 weeks after transplanting, height and leaf number were recorded before aboveground plant material was collected, dried, and weighed for biomass. Game cameras were utilized intermittently to identify any key herbivores of poison ivy.

#### 4.3.4. Analysis

Survival was modeled using a logistic regression with initial height as a covariate, exclusion treatment, and block nested within site. To evaluate the effect of herbivory exclusion on plant vigor, final plant height, and natural log-transformed aboveground biomass were modeled using least squares regression with the main effects of exclusion treatment and were block nested within site. In both models, contrasts were used to evaluate differences in means between the levels of herbivore exclusion. Disease incidence was modeled separately using a logistic regression with firth-adjusted maximum likelihood with exclusion treatment, and block nested within site as the main effects with initial height included as a covariate.

The incidence of herbivory occurring in 2018 was modeled using a logistic regression to test whether plants that had experienced herbivory in 2017 were more or less likely to experience herbivory in the following year, which was included as a binomial predictor. Further, exclusion treatment, initial height (covariate), and block nested within site were included. This model did not include plants that suffered from a disease, as our focus was limited to herbivory. All analyses were performed using the JMP 13 Pro (SAS Institute Inc., Cary, NC, USA).

Additionally, herbivory extent in 2018 was classified into seven levels according to the following—(0) no herbivory, (1) low, classified as 1–25% of a leaf removed; (2) moderate, as 25–50% of a leaf removed; (3) high, as 50–99% of a leaf removed, (4) very high, as 1 full leaf removed, (5) severe, as 2 or more full leaves removed, or (6) death of the plant. Herbivory extent was treated as an ordinal response and fitted using a cumulative link model using a logit link, with exclusion treatment and block nested within site as fixed effects. This model did not include plants that suffered from disease. This analysis was conducted in R (3.4.3: R Core Team, 2017) using the *ordinal* package.

## Figures and Tables

**Table 1:** Summary statistics for each treatment on poison ivy (*Toxicodendron radicans*) seedling emergence in the first year and survival over the course of the three-year study, as well as summary statistics for site characteristics on percent seedling emergence per plot in the first year. Treatment was for “treated” versus “untreated” drupes, while habitats were forest interior and forest edge.

	Emergence		Survival		Percent Emergence		
	X^2^	*p*	X^2^	*p*	SS	F	*p*
Habitat	105.3	**<0.001**	11.5	**<0.001**	–	–	–
Treatment	90.4	**<0.001**	3.2	0.076	1.03	31.24	**<0.001**
Site	60.2	**<0.001**	17	**0.002**	0.53	4.05	**0.005**
Habitat x Treatment	2.5	0.116	0.6	0.446	–	–	–
% Bare Ground	–	–	–	–	0.4	12.04	**<0.001**
% Soil Moisture	–	–	–	–	0.06	1.71	0.194
Soil pH	–	–	–	–	0.23	7.02	**0.01**

Sums of squares (SS), and F-statistics are shown for the general linear models and Χ^2^-statistic for logistic regressions. Excluded terms are denoted by (–). Bold model terms are considered significant at *p* < 0.05.

**Table 2:** Summary statistics for each treatment on the incidence of herbivory and disease, and survival of poison ivy (*Toxicodendron radicans*) plants over the course of the two-year herbivore exclusion study. Treatments included no exclusion, large mammal, small and large mammal exclusion, and full exclusion.

	Incidence of Herbivory		Incidence of Disease		Survival	
	X^2^	*p*	X^2^	*p*	X^2^	*p*
Treatment	1.13	0.770	0.71	0.871	3.59	0.309
Block [Site]	6.83	0.337	14.49	**0.025**	8.55	0.200
Site	27.26	**<0.001**	5.29	**0.071**	5.84	0.054
Initial Height	9.80	**0.002**	0.57	0.450	0.54	0.461
Herbivory in 2017	0.14	0.706	–	–	–	–

Excluded terms denoted by (–). Bold model terms are considered significant at *p* < 0.05.

**Table 3:** Contrasts (B) * of each exclusion treatment relative to the full exclusion treatment on herbivory extent observed on poison ivy (*Toxicodendron radicans*) plants in year two of the herbivore exclusion experiment (z is the test statistic).

	Herbivory Extent		
	B ± se	z	*p*
No Exclusion	1.0 ± 0.4	2.31	**0.021**
Large Vertebrate Exclusion	0.6 ± 0.4	1.29	0.198
Large/Small Vertebrate Exclusion	0.4 ± 0.4	0.8	0.426

* A positive contrast indicates that plants in this treatment experienced more severe herbivory than those in the full exclusion treatment. Bold model terms are considered significant at *p* < 0.05.

**Table 4:** Summary statistics for exclusion treatment (no exclusion; large mammal, small and large mammal exclusion; and full exclusion) on plant aboveground biomass (natural log transformed) and final plant height for poison ivy (*Toxicodendron radicans*) seedlings.

	Log Biomass			Height		
	SS	F	*p*	SS	F	*p*
Treatment	4.41	2.6	**0.055**	213.7	4.59	**0.004**
Site	1.9	1.68	0.191	154.53	4.97	**0.008**
Initial Height	9.09	16.04	**<0.001**	702.76	45.21	**<0.001**
Block [Site]	3.75	1.1	0.363	141.81	1.52	0.176

Bold model terms are considered significant at *p* < 0.05.

## Data Availability

The data presented in this study are openly available in [University Libraries, Virginia Tech] at [doi:10.7294/p5f2-2c10] reference number [Dickinson, C., Jelesko, J., Barney, J. Habitat suitability and establishment limitations of a problematic liana. 2021].
